# Reprogramming of fatty acid metabolism in cancer

**DOI:** 10.1038/s41416-019-0650-z

**Published:** 2019-12-10

**Authors:** Nikos Koundouros, George Poulogiannis

**Affiliations:** 1 Signalling and Cancer Metabolism Team, Division of Cancer Biology, The Institute of Cancer Research, 237 Fulham Road, London, SW3 6JB UK; 2 Division of Computational and Systems Medicine, Department of Surgery and Cancer, Imperial College London, London, SW7 2AZ UK

**Keywords:** Cancer metabolism, Lipid signalling

## Abstract

A common feature of cancer cells is their ability to rewire their metabolism to sustain the production of ATP and macromolecules needed for cell growth, division and survival. In particular, the importance of altered fatty acid metabolism in cancer has received renewed interest as, aside their principal role as structural components of the membrane matrix, they are important secondary messengers, and can also serve as fuel sources for energy production. In this review, we will examine the mechanisms through which cancer cells rewire their fatty acid metabolism with a focus on four main areas of research. (1) The role of de novo synthesis and exogenous uptake in the cellular pool of fatty acids. (2) The mechanisms through which molecular heterogeneity and oncogenic signal transduction pathways, such as PI3K–AKT–mTOR signalling, regulate fatty acid metabolism. (3) The role of fatty acids as essential mediators of cancer progression and metastasis, through remodelling of the tumour microenvironment. (4) Therapeutic strategies and considerations for successfully targeting fatty acid metabolism in cancer. Further research focusing on the complex interplay between oncogenic signalling and dysregulated fatty acid metabolism holds great promise to uncover novel metabolic vulnerabilities and improve the efficacy of targeted therapies.

## Background

**Fig. 1:**
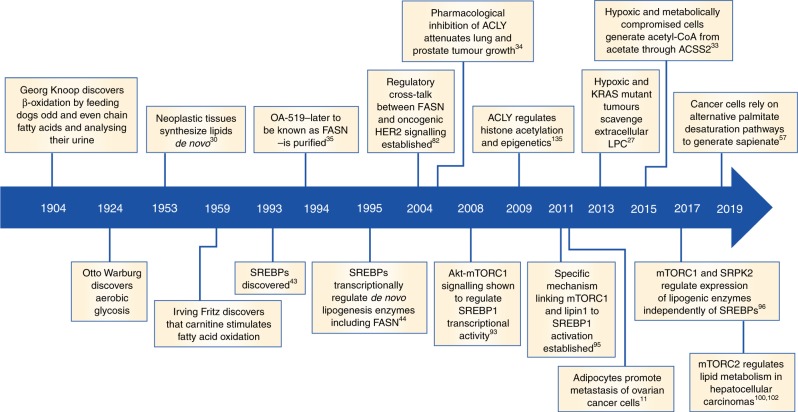
Major discoveries in lipid research. Seminal studies demonstrating the importance of dysregulated fatty acid metabolism in cancer. FASN, fatty acid synthase; SREBPs, sterol regulatory element-binding protein; ACLY, ATP–citrate lyase; mTORC1, mammalian target of rapamycin complex 1; mTORC2, mammalian target of rapamycin complex 2.

Fatty acids (FAs) are the main building blocks of several lipid species, including phospholipids, sphingolipids and triglycerides, and are composed of a carboxylic acid group and a hydrocarbon chain of varying carbon lengths and degrees of desaturation. They can be funnelled into various metabolic pathways to synthesise more complex lipid species, including diacylglycerides (DAGs) and triacylglycerides (TAGs), or converted into phosphoglycerides, such as phosphatidic acid (PA), phosphatidylethanolamine (PE) and phosphatidylserine (PS).^1^ Consequently, FAs contribute to the vast structural diversity of the cellular lipid pool, which in turn serves to regulate several biochemical processes in normal cells. These include synthesis of biological membranes and the modulation of their fluidity, functioning as secondary messengers in signalling pathways to maintain homoeostasis, and serving as a form of energy storage in animals.^2^ More than 100 years of research have provided tremendous insights into the integral role of FAs in tumorigenesis. Examples include the increased reliance of cancer cells on de novo biosynthesis and exogenous FA uptake to not only sustain their rapid proliferative rate, but also provide an essential energy source during conditions of metabolic stress (Fig. 1).^3^ In this review, we will explore how cancer cells sustain their FA metabolism in the context of a metabolically dynamic tumour microenvironment, and oncogenic signalling to support tumorigenesis and cancer progression. By considering the interplay of the aforementioned processes, we present opportunities to exploit metabolic dependencies as viable therapeutic options to tackle cancer pathogenesis.

## How do cancer cells obtain fatty acids?

In mammalian cells, FAs can either be obtained through direct exogenous uptake from the surrounding microenvironment or synthesised de novo by using nutrients, such as glucose or glutamine. It is widely accepted that a metabolic hallmark of cancer cells is lipidomic remodelling, which broadly encompasses alterations in FA transport, de novo lipogenesis, storage as lipid droplets (LDs) and β-oxidation to generate ATP.^4^ However, the specific mechanisms driving particular lipid phenotypes are nuanced, and may be dependent on tumour type or molecular sub-classifications.

### Exogenous uptake of fatty acids allows for metabolic flexibility in cancer cells

**Fig. 2:**
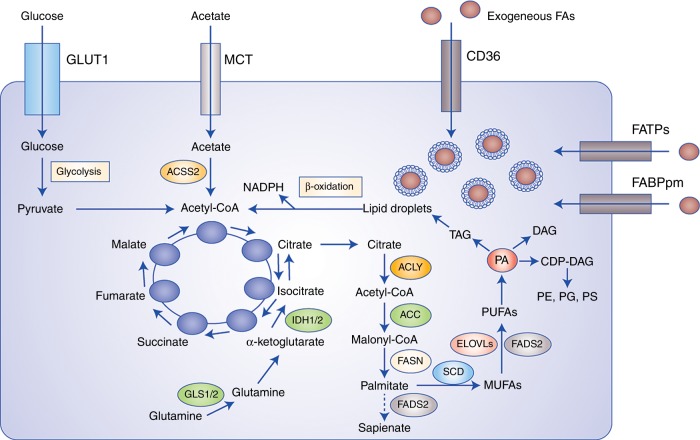
Cancer cells obtain fatty acids (FAs) from de novo lipogenesis and exogenous uptake. The exogenous uptake of FAs from the surrounding microenvironment is facilitated by specialised transporters, including CD36, FATPs and FABPpm. FAs and their synthetic products can be subsequently stored as LDs, and used for NADPH and acetyl-CoA production through β-oxidation. In terms of carbon sources for de novo lipogenesis, cancer cells rely on glucose, glutamine and acetate to synthesise citrate. Palmitate is ultimately generated from citrate through the enzymatic activities of ACLY, ACC and FASN, and can subsequently be desaturated and elongated to form a diverse group of lipid species. An alternative pathway for palmitate desaturation exists, which generates sapienate through FADS2, instead of palmitoleate. Abbreviations: GLUT1, glucose transporter 1; MCT, monocarboxylate transporter; CD36, cluster of differentiation 36; FATPs, fatty acid transport proteins; FABPpm, fatty acid-binding protein; GLS, glutaminase; IDH1/2, isocitrate dehydrogenase; ACLY, ATP–citrate lyase; ACSS2, acyl-CoA synthetase short-chain family member 2; ACC, acetyl-CoA carboxylase; FASN, fatty acid synthase; MUFAs, monounsaturated fatty acids; PUFAs, polyunsaturated fatty acids; SCD, stearoyl-CoA desaturase-1; FADS2, fatty acid desaturase 2; ELOVLs, elongation of very long-chain fatty acid protein; PA, phosphatidic acid; TAG, triacylglycerol; DAG, diacylglycerol; PE, phosphatidylethanolamine; PG, phosphatidylglycerol; PS; phosphatidylserine.

In terms of exogenous FA uptake, specialised transporters are required to facilitate efficient movement across the plasma membrane. The most well characterised of these include CD36, also known as fatty acid translocase (FAT), fatty acid transport protein family (FATPs), also known as solute carrier protein family 27 (SLC27) and plasma membrane fatty acid-binding proteins (FABPpm), all of which display increased gene and protein expression in tumours (Fig. 2).^5^ In particular, high CD36 expression has been correlated with poor prognosis across several tumour types, including breast, ovarian, gastric and prostate.^6,7^ In the case of highly aggressive *Pten*^*−/*−^ prostate cancers, CD36 promotes increased FA uptake and storage in LDs, as well as significant alterations in lipid composition encompassing elevations in acyl-carnitines (ACs), monoacylglycerols (MAGs) and other lysophospholipids, all of which are products of FA oxidation (FAO).^8^ Notably, deletion of *Cd36* is sufficient to rescue the aforementioned phenotypes and mitigate tumour growth, indicating that this FA transporter is integral for promoting the lipidomic remodelling of *Pten-*deficient prostate cancers.^8^ Collectively, these findings demonstrate that CD36 plays a key role in tumour microenvironment metabolic crosstalk, ultimately shifting the dependency of tumour cells towards exogenous lipid uptake.

An intriguing observation during the metastatic dissemination of breast, prostate and ovarian cancer cells is their preferential homing to adipocyte tissue located in periglandular regions and the visceral omentum.^9^ It has long been appreciated that adipose tissue is implicated in metabolic syndromes such as obesity, and functions as an endocrine system that secretes growth factors, cytokines and free FAs following lipolysis.^10^ In this respect, co-culture, as opposed to isolated, cell systems have been instrumental in our understanding of the impact of adipocytes on the FA metabolism of cancer cells.^11^ Importantly, these seminal studies have demonstrated that the migration and proliferation of human ovarian cancer cells are significantly increased following co-culture either directly with human-derived omentum adipocytes, or their conditioned media.^11^ In these models, adipocytes were shown to activate endogenous lipolysis of triglycerides to produce free FAs that could be subsequently secreted and taken up by metastatic cells overexpressing FABP4.^11^ The transfer of FAs from surrounding adipocytes to metastatic ovarian cancer cells also potentiated AMP-activated protein kinase (AMPK) signalling in the latter, culminating in increased β-oxidation through carnitine palmitoyltransferase 1 (CPT1) and acyl-CoA oxidase 1 activation.^11^ The overexpression of the FA translocase CD36 is also essential for driving the progression of ovarian cancer, and this is largely mediated through rapid exogenous uptake of long-chain FAs and cholesterol that likewise, are obtained from the adipocytes in the microenvironment.^7^

A major implication of elevated uptake of exogenous FAs is their subsequent storage in LDs, which are cytoplasmic organelles that sequester excess FAs in the form of TAGs and sterol esters.^12^ Consequently, the accumulation of LDs in cancer cells is used not only to maintain lipid homoeostasis and prevent lipotoxicity, but also to provide a valuable source of ATP and NADPH during conditions of metabolic stress (Fig. 2).^12,13^ This is largely achieved through β-oxidation of stored lipids, leading to the production of acetyl-CoA through oxidative degradation of FAs.^14,15^ The acetyl-CoA produced from each round of β-oxidation can subsequently enter the tricarboxylic acid (TCA) cycle to generate NADH and FADH2 for the electron transport chain, ultimately leading to the synthesis of approximately six times more ATP than oxidation of carbohydrates.^14^ Moreover, the oxidation of citrate derived from acetyl-CoA by isocitrate dehydrogenase 1 (IDH1) is one of the main sources of cellular NADPH production.^16^ Thus, β-oxidation of LDs provides sufficient ATP to fuel the metastatic cascade, and generates NADPH that is essential for anabolic metabolism and detoxification of reactive oxygen species (ROS).^17–20^ This is particularly relevant for hypoxic cells, which have elevated FA uptake and accumulation of LDs following hypoxia-inducible factor (HIF)-1α-dependent expression of FABP3 and FABP7.^21^ The oxidation of triglycerides stored in LDs provides sufficient ATP to facilitate the recovery of breast cancer and glioblastoma cells during reoxygenation, whilst the increase in NADPH levels serves to protect against ROS toxicity.^21^ Indeed, knockdown of FABP3 and FABP7 decreases the growth of U87 glioblastoma tumours in vivo, an effect largely attributable to reduced FA uptake and inhibition of LD formation.^21^

On a broader level, delineating the mechanisms driving interactions between cancer cells and adipocytes has provided fundamental insights into how obesity contributes to tumour initiation and progression. This is particularly relevant for renal, gastric, breast and colon cancers that preferentially grow in adipocyte-rich environments.^10^ It is noteworthy that adipose tissue associated with obesity recapitulates a state of persistent inflammation characterised by the secretion of tumour necrosis factor (TNF)-α, interleukin (IL)-6 and IL-8, as well as production of vascular endothelial growth factor (VEGF), prostaglandins and leukotrienes by activated macrophages.^22^ Thus, adipose cells can function as active mediators of endocrine and paracrine signalling, which in turn support the crosstalk between adiposity and cancer cell FA metabolism.^23^ Indeed, this reciprocal crosstalk perpetuates an interaction network in which secreted adipokines stimulate cancer cells to release exosomes containing pro-lipolytic factors such as miRNA-144 and miRNA-126, which in turn promote lipolysis in adjacent adipocytes through activation of AMPK signalling and induction of autophagy.^9,24^ Ultimately, enhanced lipolysis and release of free FAs fundamentally alters the metabolic dependencies of migrating cancer cells, shifting their reliance towards exogenous lipid uptake and β-oxidation for energy supply.^24,25^ These findings also point to a rationale for developing therapies targeting the tumour microenvironment through inhibition of adipocyte lipolysis, thereby reducing the availability of free lipids for cancer cells.^26^

The uptake and scavenging of extracellular FAs also provides an important compensatory mechanism for cancer cells to sustain their lipid demands under conditions of metabolic stress. For instance, the flux from glucose to acetyl-CoA decreases under hypoxic conditions, and so does the conversion of saturated FAs into monounsaturated FAs, as it is regulated by the oxygen-consuming enzyme stearoyl-CoA desaturase-1 (SCD-1). Consequently, hypoxic cells display increased uptake of exogenous lysophospholipids, such as lysophosphatidylcholine (LPC), in order to sustain their proliferation and survival.^27^ The regulation of exogenous lipid uptake under hypoxia largely occurs through the HIF-dependent overexpression of lipid-binding proteins. For instance, FABP4 is a transcriptional target of HIF1α that facilitates extracellular scavenging of long-chain unsaturated lysophospholipids, including LPCs, lysophosphatidylethanolamines (LPEs) and lysophosphatidylglycerols (LPGs) that can be used as a nutrient source under conditions of metabolic stress.^27,28^ Interestingly, this phenotype of increased FA scavenging is also recapitulated under normoxic conditions following oncogenic Ras activation, and is accompanied by reduced oxygen consumption, elevated citrate synthesis from reductive carboxylation and a consequent independence from SCD-1 to derive unsaturated FAs.^27^ Taken together, these results demonstrate that whilst changes in the microenvironment conditions or oncogenic activation of signalling pathways confer resistance to SCD-1 inhibitors, they might open novel opportunities for therapy by increasing the reliance of cancer cells on FA uptake.

### De novo lipogenesis allows for the synthesis of a diverse group of fatty acids

De novo lipogenesis is the process through which carbon atoms derived from carbohydrates such as glucose and amino acids including glutamine are converted into FAs.^29^ In normal tissue, de novo lipogenesis is restricted to hepatocytes and adipocytes; however, cancer cells may also reactivate this anabolic pathway even in the presence of exogenous lipid sources.^3,30^ The main substrate for FA synthesis is cytoplasmic acetyl-CoA that can either be derived from citrate or acetate.^3^ Carbons from glucose or glutamine contribute to citrate production either from the oxidation of pyruvate in the TCA cycle or through reductive carboxylation, respectively.^31,32^ In addition, under conditions of metabolic stress such as hypoxia or lipid depletion, cancer cells upregulate acetyl-CoA synthetase 2 (ACSS2) in order to generate acetyl-CoA from acetate.^33^ Citrate is converted into acetyl-CoA by ATP–citrate lyase (ACLY), which is the substrate for the carboxylating enzymes acetyl-CoA carboxylases (ACCs).^26,34^ The irreversible carboxylation of acetyl-CoA into malonyl-CoA is the rate-limiting step of de novo lipogenesis, and it is the condensation of seven malonyl-CoA molecules and one molecule of acetyl-CoA catalysed by fatty acid synthase (FASN), which ultimately produces the saturated 16-carbon FA palmitate (FA16:0).^26,35^ Subsequent desaturation of palmitate by stearoyl-CoA desaturase (SCD) produces monounsaturated FAs with a double bond at position Δ9, whilst elongation by FA elongases, such as ELOVL6, adds two-carbon groups to palmitate in order to form the saturated FA stearate (Fig. 2).^26,36,37^

The regulation of de novo lipogenesis occurs largely at the transcriptional level through the activation of sterol regulatory element-binding proteins (SREBPs), of which three main transcription factors exist: SREBP1a and SREBP1c arising from the alternative splicing of *SREBPF1*, and SREBP2 encoded by the *SREBPF2* gene.^38^ SREBPs are initially found as inactive 125-kDa precursors, bound to the endoplasmic reticulum (ER). At saturated concentrations of intracellular cholesterol, insulin-induced genes (INSIGs) bind to SREBP-cleavage-activating proteins (SCAPs) and localise the SREBP precursors to the ER.^39,40^ Conversely, under conditions of low cholesterol levels, SCAPs facilitate the translocation of ER-bound SREBPs to the Golgi, where the transcription factor is subsequently cleaved by membrane-bound transcription factor site 1 proteases (MBTPS1 and MBPTS2) to release the active N terminus.^41,42^ Ultimately, it is the N-terminal fragment that translocates to the nucleus and induces the transcription of genes containing sterol regulatory elements (SREs), such as *FASN, ACLY* and *ACC*.^26,43,44^

One of the main advantages for cancer cells to sustain higher de novo lipogenesis of FAs is their flexibility to shunt them into different biosynthetic pathways to generate a diverse cellular pool of lipid species with distinct functions. Indeed, upregulation of lipid synthesis, followed by downstream elongation and desaturation pathways, has been shown to be sufficient to produce all FAs from the nutrients, such as glucose, glutamine and acetate, which are required for adipocyte differentiation.^45^ Palmitate (FA16:0) is the main product of de novo lipogenesis, and can be elongated and desaturated through the activity of SCD, ELOVLs and FADs to produce additional FA species including stearate (FA18:0) and oleate (FA18:1) (Fig. 2).^46^ These FAs can be subsequently used for the production of more complex lipids. Notably, oleate can directly feed into PA synthesis through the enzymatic activities of glycerol-3-phosphate acyltransferase 1 (GPAT1) and acyl-CoA:LPA acyltransferase (LPAT), as well as being incorporated into TAGs for storage in a GPAT1-dependent fashion.^47–49^ With respect to PA, this class of phospholipid not only serves important structural and signalling roles, but is also one of the main substrates for DAG synthesis by Lipin1–3 and AGPAT, as well as contributing to the biosynthesis of complex glycerolipids.^50,51^ For instance, PA can be condensed with CTP through a reaction catalysed by CDP–DAG synthase (CDS) to generate CDP–DAG.^52,53^ Importantly, CDP–DAG is the main precursor for the de novo synthesis of complex glycerolipids including phosphatidylinositols (PtdIns), phosphatidylserines (PSs), phosphatidylglycerols (PGs) and phosphatidylcholines (PCs) (Fig. 2).^54^

More recently, studies have begun to elucidate the various compensatory pathways implicated in FA metabolism that cancer cells exploit to increase their adaptability. This has been most extensively studied with the SCD enzymes, which were previously thought to be the only desaturases that generate monounsaturated FAs from palmitate. SCDs are necessary for tumorigenesis as they regulate the cellular pool of unsaturated FAs that later serve as building blocks for phosphoglycerides, phosphoinositides, eicosanoids and sphingolipids. As a result, they constitute an attractive target for therapeutic intervention; however, inhibitors targeting these metabolic enzymes have only shown modest effects.^55,56^ This suggests that cancer cells may rely on alternative desaturation pathways to generate functionally useful lipid species, thus relieving their dependence on canonical SCD-mediated desaturation. Indeed, FADS2 has been shown to play a dominant role in FA desaturation in cancer cell lines and primary tumours that are resistant to SCD inhibitors.^57^ Of note, these cells exploit an alternative pathway involving the FADS2-dependent desaturation of palmitate to sapienate (*cis*-6-C16:1) to support their membrane synthesis during proliferation.^57^

## The context-dependent regulation of lipid metabolism: convergence of molecular heterogeneity and oncogenic signalling

Several metabolic processes in cancer cells are directly regulated by oncogenes and tumour suppressors. For instance, mutant *KRAS* enhances glycolysis in pancreatic cancer cells through upregulation of the genes encoding hexokinase 1/2 (*Hk1* and *Hk2*, respectively), and redirects glutamine flux to malate for the production of pyruvate and reducing power in the form of NADPH.^58,59^ Moreover, amplification of the MYC oncogene drives increased glutamine metabolism and anaplerosis by transcriptionally activating mitochondrial glutaminase (GLS1) and the SLC1A5 glutamine transporter.^60,61^ Hyperactivation of the phosphoinositide 3-kinase and AKT (PI3K–AKT) pathway has also been implicated in the rewiring of specific metabolic processes, including increased glucose uptake through stabilisation of glucose transporter 1 (GLUT1), enhanced glutamine anaplerosis via activation of glutamate pyruvate transaminase 2 (GPT2) and remodelling of the cellular lipidome.^62–64^ It is also becoming increasingly appreciated that the complex regulatory networks involved in FA metabolism must be considered in specific molecular and metabolic contexts. These include the impact of molecular heterogeneity, as exemplified by the intrinsic subtypes of breast cancer, as well as the oncogenic processes that drive malignant transformation in different cancers.

### Molecular heterogeneity and lipid reprogramming

Gene expression analyses have long suggested that upregulation of several enzymes involved in lipid metabolism is a near-universal metabolic hallmark of cancer cells.^4,65^ However, more nuanced observations from these genomic studies highlight differences in the expression of lipid enzymes across different tumour types and molecular sub-classifications.^4^ For instance, long-chain acyl-CoA synthetase 3 (ACSL3) is overexpressed in androgen-dependent cancers, such as prostate tumours, where it activates cholesterol synthesis and steroidogenesis, but it is downregulated in triple-negative breast cancers.^66,67^ Furthermore, enzymes involved in β-oxidation such as α-methylacyl-CoA racemase (AMACR) and CPT1B are specifically overexpressed in colorectal, hepatic and prostate cancers, whilst CPT1A is upregulated in breast cancer.^4,68^ The upregulation of AMACR in prostate cancer is highly significant to the extent that it is used as a bona fide biomarker for early detection and diagnosis of prostate cancer.^68^ Furthermore, it also suggests that β-oxidation, particularly of branched-chain FAs, is the main bioenergetic pathway for obtaining ATP and NADPH in prostate cancer cells.^69^ Clinically, prostate cancers display low rates of glycolysis, and are therefore poor candidates for diagnostic imaging by using fludeoxyglucose positron emission tomography (FDG-PET).^70^ The higher dependence on FAO could serve as an alternative metabolic signature for prostate cancer diagnosis, and indeed this dependency is currently being explored as a novel imaging application through the use of C11-acetate PET tracers.^70^ Thus, although dysregulated lipid metabolism is a broad feature of cancer, different tumour types may exhibit unique metabolic adaptations that contribute to the remodelling of their lipidome.

The regulatory complexity of lipid metabolism in the context of molecular heterogeneity is perhaps best exemplified in breast cancer, which comprises multiple different subtypes characterised by unique hormone/growth-factor receptor expression and genetic profiles.^71^ Comparative mRNA expression analyses between receptor-positive and triple-negative breast cancers (RPBC and TNBC, respectively) have revealed notable differences between these subtypes in terms of lipid procurement, storage and oxidation.^4^ Interestingly, RPBCs are associated with a gene signature encompassing elevated de novo lipogenesis, FA mobilisation and oxidation, whilst TNBCs overexpress genes involved in exogenous lipid uptake – including FABP5 and FABP7 – and storage.^4^ It is also notable that TNBCs are dependent on different ACSL isoforms when compared with RPBCs to mobilise FAs that have been stored in LDs.^4^ Specifically, overexpression of ACSL4 in TNBCs is associated with the enhanced metabolism of arachidonic acid to arachidonyl-CoA that can be used as a substrate for cyclo-oxygenase 2 (COX2) for prostaglandin synthesis.^4^ The co-ordinated synthesis of pro-inflammatory prostaglandins from LDs may, in turn, contribute to the more aggressive phenotypes generally observed in TNBCs compared with RPBCs.^4,72^

Although gene expression analyses may not necessarily provide a direct reflection of enzyme activity or dependencies on specific metabolic pathways, studies have validated unique lipid-associated genetic signatures that can be used to guide therapeutic intervention.^67,73^ For instance, a subset of TNBCs with MYC overexpression have been shown to upregulate several genes involved in β-oxidation, such as *PGC1α*, *CPT1B* and *CDCP1*, whilst downregulating *FASN* and *ACACB*.^73^ Importantly, a genetic signature associated with FAO contributes to the aggressiveness and poor clinical outcome of MYC-high TNBCs, suggesting that it is an essential bioenergetics pathway of these tumours.^73^ Indeed, inhibition of CPT1 and consequently FAO with etomoxir significantly reduces the primary tumour growth of MYC-high, but not MYC-low, TNBCs or RPBCs.^73^ The potential to exploit the metabolic dependency of TNBCs on β-oxidation has also been demonstrated for the treatment of metastatic disease, with the inhibition of CUB-domain-containing protein 1 (CDCP1) significantly impairing the capacity of TNBCs to oxidise FAs stored in LDs during migration and metastasis.^67^ Overall, rather than considering dysregulated lipid homoeostasis as a general feature of cancer cells, it is important to understand the molecular subtype, tissue and the overall tumour microenvironment context of such changes. This will undoubtedly lead to better stratification methods and targeted application of metabolic inhibitors that block specific pathways involved in lipid metabolism.

### Regulation of lipid metabolism by oncogenic signalling

Oncogenic signalling pathways can directly regulate metabolic enzymes involved in lipid metabolism, and are therefore integral in shaping the tumour lipidome. The most frequently dysregulated signalling pathway in human cancers is PI3K–AKT signalling, which can be activated through the stimulation of growth-factor receptor tyrosine kinases (RTKs) including human epidermal growth-factor receptor 2 (HER2) and the insulin receptor, or acquirement of oncogenic mutations in *PIK3CA*, the gene encoding the p110α catalytic subunit of class I PI3Ks.^74–77^ *PIK3CA* is, in fact, one of the most commonly mutated genes in carcinomas, with up to a third of all human cancers and 40% of breast cancers carrying gain-of-function mutations.^78^ There have been several extensive reviews on the specific nodes that constitute PI3K signalling,^74,75,79^ and their oncogenic consequences in terms of promoting growth, proliferation and survival, hence these concepts are only briefly introduced here.

**Fig. 3:**
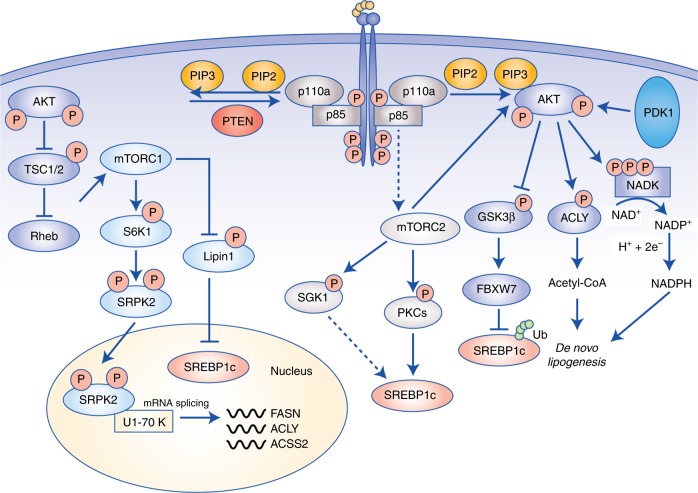
Regulation of lipid metabolism by PI3K–mTOR signalling. PI3K signalling is the most frequently dysregulated pathway in cancer, and stimulates growth, proliferation and survival. Activation of receptor tyrosine kinases recruits PI3Kα to the plasma membrane where it phosphorylates PIP_2_ to PIP_3_. AKT binds to PIP_3_, allowing activation by PDK1 and mTORC2. AKT directly promotes lipogenesis by stabilising SREBP1c through inhibition of GSK3β, activation of ACLY to generate acetyl-CoA and phosphorylation of NADK to produce NADP^+^ for NADPH synthesis. PI3K signalling is also closely linked to mTORC1 and mTORC2. mTORC1 regulates lipogenesis through inhibition of lipin-1, which is a negative regulator of nuclear SREBP1c, and activation of the splicing factor SRPK2, thereby promoting the expression of lipogenic enzymes, including ACLY, FASN and ACSS2. Finally, mTORC2 activation supports lipogenesis through AKT-dependent and -independent mechanisms, with the latter encompassing phosphorylation of SGK1 and PKCs, and subsequent activation of SREBP1c. Abbreviations: PIP_3_, phosphatidylinositol (3,4,5)-trisphosphate; PIP_2_, phosphatidylinositol (4,5)-bisphosphate; mTORC, mammalian target of rapamycin complex; SREBP, sterol regulatory element-binding protein; SGK, serum- and glucocorticoid-induced protein kinase 1; PKC, protein kinase C; GSK3, glycogen synthase kinase; FBXW7, F-Box and WD repeat domain containing 7; ACLY, ATP–citrate lyase, PDK1, phosphoinositide-dependent kinase 1; NADK, NAD^+^ kinase; NAD^+^, nicotinamide adenine dinucleotide; NADP^+^, nicotinamide adenine dinucleotide phosphate; SRPK2; SR-protein-specific kinase 2; S6K1, ribosomal protein S6 kinase β-1; FASN, fatty acid synthase; ACC, acyl-CoA synthetase short-chain family member 2.

HER2-amplified breast cancers are closely associated with hyperactivation of PI3K signalling, with more than 80% of tumours displaying increased phosphorylation of AKT on Ser^473^ and Thr^308^.^80^ Furthermore, an important feature of HER2-positive tumours that contributes to their aggressiveness is sustained upregulation of de novo lipogenesis.^81^ Indeed, overexpression of HER2 in non-transformed epithelial cells induces a lipogenic phenotype dependent on FASN activation that is reminiscent of cancer cells, whilst inhibition of HER2 or de novo lipogenesis ablates oncogenic activity and induces apoptosis.^82^ This suggests that oncogenic signalling downstream of HER2 may activate several complementary pathways that converge on increased lipogenesis. Activation of AKT contributes to two essential processes for de novo lipid synthesis: the shuttling of metabolic intermediates to provide carbon sources for anabolism, and the synthesis of reducing equivalents in the form of NADPH to fuel lipogenesis.^83^ For instance, AKT can directly phosphorylate and activate ACLY, thus increasing acetyl-CoA synthesis.^84^ Moreover, NADPH is an essential cofactor for anabolic metabolism, and specifically for the condensation reaction of acetyl-CoA and malonyl-CoA catalysed by FASN.^85^ AKT can indirectly promote NADPH production by activating the nuclear factor-like 2 (Nrf2) transcription factor,^20,86^ leading to the transcription of genes involved in NADPH synthesis, including 6-phosphogluconate dehydrogenase (6PGD), glucose-6-phosphate dehydrogenase (G6PD) and malic enzyme 1 (ME1).^87,88^ More recently, AKT has been shown to directly contribute to the cellular pool of NADPH by acutely activating nicotinamide adenine dinucleotide kinase (NADK), resulting in increased nicotinamide adenine dinucleotide phosphate (NADP^+^) production.^89^ Mechanistically, AKT-mediated phosphorylation of NADK at Ser^44^, Ser^46^ and Ser^48^ within the N-terminal domain maintains NADK in an active state by preventing its autoinhibition.^89^ Importantly, NADK is the only enzyme in mammalian cells that converts NAD^+^ into NADP^+^, the latter of which can be reduced to NADPH to sustain de novo lipogenesis (Fig. 3).^89^

Intimately linked with PI3K activation is signalling through the mammalian target of rapamycin (mTOR) complexes. mTOR is a serine/threonine protein kinase that is the predominant catalytic subunit of the protein complex mTOR complex 1 (mTORC1) and mTOR complex 2 (mTORC2).^90^ Both are multiprotein complexes, and are distinguished mainly through their associations of either Raptor (regulatory protein associated with mTOR) in mTORC1, or Rictor (rapamycin-insensitive companion of mTOR) in mTORC2.^90^ AKT is a potent activator of mTORC1 by phosphorylating and inactivating its main negative regulators tuberous sclerosis complex components 1 and 2 (TSC1/2).^90^ Several metabolic processes are activated by mTORC1, including oxidative phosphorylation by promoting mitochondrial biosynthesis,^91^ sustaining de novo nucleotide synthesis^92^ and lipogenesis.^93^ The processing of the lipogenesis transcriptional regulator SREBP1 into its mature active form is strongly influenced by PI3K–AKT–mTORC1-dependent mechanisms; hence, the expression of key lipogenic enzymes, such as *FASN*, *ACC1* and *ACLY* is suppressed following mTORC1 inhibition by acute rapamycin inhibition or Raptor knockdown.^93,94^ One of these mechanisms involves the mTORC1-dependent regulation of lipin-1.^95^ Interestingly, mTORC1 directly phosphorylates and inactivates lipin-1 leading to its sequestration in the cytoplasm.^95^ Upon mTORC1 inhibition, active lipin-1 translocates to the nucleus and induces significant nuclear reshaping, culminating in reduced SREBP-transcriptional activity (Fig. 3).^95^ Although not fully elucidated, this regulation may be dependent on the lipid phosphatase activity of lipin-1 that specifically acts on PAs: it is, therefore, conceivable that modulation of the lipid architecture in the nucleus and subsequent alteration of the nuclear lamina may directly affect SREBP activity.^95^ mTORC1 also regulates the expression of lipogenic genes independently of SREBPs.^96^ Mechanistically, this requires the mTORC1–S6 kinase 1 (S6K1)–serine/arginine protein kinase 2 (SRPK2) activation of the U1 small nuclear ribonucleoprotein 70 kDa (U1-70K), leading to increased mRNA splicing of genes involved in de novo lipogenesis such as *FASN, ACLY* and *ACSS2* (Fig. 3).^96^ Notably, inhibition of mTORC1 with Torin-1 reduces spliceosome formation, and knockdown of SRPK2 significantly attenuates mRNA stability and expression of the aforementioned lipogenic enzymes, resulting in reduced tumour growth in vivo.^96^

It is noteworthy that several oncogenes converge on mTORC1 to promote lipogenesis. One of the most relevant in the context of tumorigenesis is activation of Ras.^97^ KRAS in particular is frequently mutated in colorectal and non-small-cell lung carcinomas, with the G12V missense accounting for the majority of detected mutations.^97^ Consequent activation of extracellular signal-regulated kinase 1/2 (ERK1/2) activates mTORC1 through inhibition of TSC1/2, and contributes to lipidomic rewiring encompassing elevated synthesis of glycerophospholipids and FAs including palmitate, oleic acid and docosahexaenoic acid in lung cancers.^94,98,99^ Furthermore, KRAS^G12V^ also contributes to increased incorporation of acetate into endogenously synthesised lipids, potentially suggesting a dependence on ACSS2 to synthesise acetyl-CoA for de novo lipogenesis.^94^

In addition to mTORC1, hyperactivation of PI3K signalling can also induce mTORC2 activity, and although less well characterised than mTORC1, it is becoming increasingly appreciated that mTORC2 is an important mediator of metabolic reprogramming in cancer cells. mTORC2 functions as a pivotal signalling hub for driving FA metabolism, and this is mediated not only through the activation of downstream AGC kinases, including AKT, serum- and glucocorticoid-regulated kinases (SGKs) and protein kinase Cs (PKCs), but also through reciprocal stimulation of mTORC2 by AKT following phosphorylation of SIN1 on Thr^86^.^79,100,101^ In support of a broad role of mTORC2 at regulating whole-body metabolism, *Rictor* knockout causes insulin insensitivity characterised by reduced glucose uptake in hepatocytes and overall attenuation of insulin-stimulated de novo lipogenesis through inhibition of SREBP1c maturation.^100^ An important role for mTORC2 signalling in tumorigenesis has been established in hepatocellular carcinogenesis, where it drives the progression of fatty liver disease to cancer in liver-specific *PTEN*^−/−^ and *Tsc1*^−/−^ mouse models.^102^ Through activation of SREBP1c, mTORC2 induces widespread alterations in the hepatocellular lipidome culminating in de novo synthesis of sphingolipids, glycerophospholipids and cardiolipins, which increase mitochondrial respiration.^102^ Importantly, inhibition of mTORC2, but not mTORC1, leads to a reduction in the elevated expression of de novo lipogenesis genes including *FASN*, and overall hepatocellular lipid content, culminating in attenuated growth of *PTEN*- and *Tsc1*-null hepatocellular carcinomas.^102^ These observations are particularly relevant in light of previous in vivo data suggesting that mTORC1 activation alone is insufficient to stimulate lipid synthesis without functional AKT.^103^

Independently of mTORC1, AKT promotes the stability of mature SREBP1c by inhibiting glycogen synthase kinase-3 (GSK3), which phosphorylates and promotes its ubiquitylation and proteasomal degradation (Fig. 3).^104^ In addition, by inhibiting the expression of Insig2, AKT positively regulates the translocation of SREBP1c from the ER membrane to the Golgi apparatus, where it can be processed into the transcriptionally active form.^103^ Therefore, it is conceivable that mTORC2 activation and its downstream signalling nodes could play a more dominant role in lipid homoeostasis than previously appreciated.^102^

An important aspect of mTORC2 signalling is its capacity to stimulate several AKT-independent compensatory signalling axes, posing significant challenges for the successful therapeutic targeting of PI3K–mTOR signalling.^105^ In addition to AKT, mTORC2 can directly phosphorylate and activate several SGK and PKC isoforms that can stimulate lipogenesis (Fig. 3). For instance, in yeast models, mTORC2 promotes ceramide synthesis by activating Ypk2, the human orthologue of which is SGK1.^106^ Although not yet comprehensively studied in human cells, the potential regulation of ceramide synthesis by mTORC2 and SGK1 could provide a compensatory pathway for sustaining glycerophospholipid synthesis in cancer independently of canonical AKT–mTORC1 activities.^106^ In terms of PKC signalling, mTORC2 phosphorylates the hydrophobic motif of several isoforms, including PKCβII, PKCε and PKCζ on Ser^660^, Ser^729^ and Thr^560^, respectively (Fig. 3).^107–109^ The regulation of cancer cell metabolism by PKCs has been characterised in the context of lipid homoeostasis and includes the PKCβ-dependent stimulation of de novo lipogenesis by activating the transcription of *SREBPF1* directly through recruitment of Sp1 to the promoter region.^110^ Moreover, the atypical PKCζ and PKCλ/ι isoforms have been shown to be the predominant mediators of hepatic lipogenesis downstream of insulin-induced PI3K signalling.^111^ The expression of *SREBPF1, FASN*, as well as glucose uptake, cholesterol and triglyceride levels, are all reduced following deletion of *Pik3r1* and *Pik3r2*.^111^ Notably, reconstituting myristoylated AKT restores hepatic glucose metabolism, whilst SREBP1c expression and lipogenesis are rescued following overexpression of PKCζ/λ.^111^ These findings indicate that PI3K signalling regulates metabolic homoeostasis in the liver through two distinct pathways: one involving AKT-dependent glucose uptake, and the second requiring PKCζ/λ to sustain lipid synthesis.^111^

It is well established that PI3K signalling regulates a myriad of cellular metabolic processes in cancer, but how these are integrated in the context of dysregulated FA metabolism is still obscure. The PI3K–AKT pathway is well-known to be the major signalling cascade contributing to the Warburg effect by directly facilitating glucose uptake and glycolysis through activation of GLUT1 and hexokinase.^112^ Furthermore, AKT has been shown to promote HIF1α mRNA translation in an mTORC1-dependent fashion even under aerobic conditions, thus contributing to uncoupling of glycolysis and mitochondrial oxidative phosphorylation through inhibition of pyruvate dehydrogenase.^113^ If one is to consider this metabolic rewiring in isolation, then it becomes counterintuitive as to how PI3K signalling promotes lipid metabolism, particularly when functional oxidative phosphorylation is required for citrate synthesis during lipogenesis and β-oxidation.

It is, therefore, necessary to re-examine the notion that cancer cells displaying elevated aerobic glycolysis must also have impaired oxidative phosphorylation. In the context of PI3K signalling, metabolic pathways contributing to both the synthesis of metabolic intermediates required for lipogenesis and promotion of mitochondrial bioenergetics are activated. For instance, glucose-6-phosphate generated during the first step of glycolysis can be shunted to the pentose phosphate pathway (PPP) to contribute in the production of NADPH, which plays a key role in the sustainment of anabolic processes and detoxification of ROS in rapidly proliferating cells.^88,114^ As discussed previously, AKT can also directly facilitate NADPH synthesis following phosphorylation and activation of NADK.^89^ Furthermore, mTORC1 and mTORC2 downstream of oncogenic PI3K promote oxidative phosphorylation through PGC-1α-dependent mitochondrial biosynthesis, and synthesis of cardiolipins, which enhance respiration and improve mitochondrial activity.^91,102^ Therefore, hyperactive PI3K signalling provides a clear metabolic advantage to cancer cells as it not only increases the synthesis of metabolic intermediates required for anabolic metabolism, but also promotes respiration to generate citrate from acetyl-CoA for de novo lipogenesis.

In general, PI3K–AKT signalling promotes lipid synthesis whilst inhibiting lipolysis and β-oxidation.^115^ However, the precise involvement of the PI3K pathway in balancing the oxidation of FAs and glucose is more complex. Insights have been provided by studies focussing on glucose and lipid homoeostasis in type 2 diabetes. Hyperinsulinaemia activates the insulin receptor and PI3K signalling, contributing to increased CD36 membrane translocation and uptake of exogenous FAs.^116^ At the onset of insulin resistance, GLUT4 expression and translocation is inhibited, leading to increased blood glucose levels, decreased glycolysis and high FA β-oxidation of intracellular lipid pools.^117^ In terms of specific mechanisms, the accumulation of FAs in response to insulin resistance may drive DAG synthesis, which can activate conventional PKC isoforms, leading to the phosphorylation of insulin receptor substrate 1 (IRS1) and consequent inhibition of PI3K–AKT signalling.^118^ Moreover, dysregulated lipid signalling could induce a negative-feedback loop initiated by the activation of mTORC1–p70S6K and its subsequent phosphorylation-induced inhibition of IRS1.^119,120^ Given that insulin resistance and obesity are becoming increasingly associated with cancer, it is conceivable that PI3K signalling is central to this phenomenon. In this context, attenuation of PI3K signalling following obesity-induced insulin resistance may shift the metabolic balance from lipid synthesis to lipolysis and β-oxidation.^115^ As previously discussed in this review, increased lipolysis particularly in adipocyte-rich microenvironments generates free FAs that can be utilised by cancer cells,^7,11^ whilst β-oxidation contributes to ATP and NADPH synthesis.^14^ Thus, the effects of PI3K signalling on lipid metabolism and tumorigenesis are paradoxical: on one hand, active PI3K signalling supports tumorigenesis through enhanced de novo lipogenesis, but on the other it inhibits lipolysis and β-oxidation, both of which are key at promoting cancer cell proliferation and metastasis. In order to reconcile the seemingly disparate regulatory mechanisms linking PI3K signalling and lipid metabolism, it may be necessary to consider whole-body metabolism and obesity as factors that dictate the dependencies of tumours for specific metabolic pathways.

### Regulation of oncogenic signalling by fatty acid metabolism

Thus far, rewiring of the tumour lipidome has been largely discussed as a consequence of hyperactive oncogenic signalling and genetic aberrations. However, it is also important to acknowledge that overexpression of several lipogenic enzymes occurs relatively early in tumorigenesis, and can be observed in both hyperplastic and preinvasive lesions.^65,121^ Therefore, it is conceivable that upregulation of de novo lipogenesis and the enzymatic network that regulates it may also dynamically and reciprocally potentiate oncogenic signalling throughout malignant transformation, rather than representing a secondary phenomenon. In support of this, mechanisms linking the bidirectional crosstalk between FASN and oestrogen receptor α (ERα) signalling have been elucidated in hormone-dependent breast cancers. In these models, both genetic and pharmacological inhibition of FASN hypersensitises ERα to oestrogen-dependent transactivation, thus leading to a synergistic induction of both oestrogen receptor element (ERE) transcriptional activity and mitogen-activated protein kinase (MAPK)–ERK signalling.^122^ Surprisingly, oestrogen stimulation following FASN inhibition has significant cytotoxic and cytostatic effects, despite activation of the seemingly pro-tumorigenic aforementioned processes.^122^ Whilst this may seem paradoxical at first, these findings highlight a novel role for FASN in modulating the thresholds required for triggering hormone receptor signalling, and consequently regulating the balance between the opposing pro-tumorigenic and anti-proliferative effects of ERα stimulation. For instance, although oestrogen signalling contributes to the upregulation of genes involved in proliferation, invasion and metastasis – such as Twist and matrix metalloproteinases 2/9 (MMP2/9) – FASN blockade leads to the nuclear accumulation of the oestradiol (E_2_) targets p21^WAF/CIP1^ and p27^Kip1^, culminating in cell-cycle inhibition and induction of cytostatic and cytotoxic effects in response to E_2_ exposure.^122,123^ Moreover, it is interesting to note that while E_2_ hyperactivates MAPK–ERK signalling following FASN inhibition, PI3K–AKT activity is attenuated.^122^ Indeed, there exists significant crosstalk between MAPK–ERK and PI3K–AKT signalling encompassing both positive- and negative-feedback loops, and this may explain at least one of the mechanisms responsible for the observed effects of FASN blockade and E_2_ exposure.^124^ In terms of cross-inhibition, ERK has been shown to phosphorylate the scaffold protein GRB2-associated-binding protein 1 (GAB1), thus compromising the recruitment of PI3K to the plasma membrane and downstream activation of AKT.^125^ Therefore, in addition to its role in de novo lipogenesis, FASN is also at the nexus of pathway integration between MAPK–ERK and PI3K–AKT pathways, and their interaction with E_2_–ERα signalling.^122^

Molecular connections have also been made between FASN and HER2 overexpression.^121^ These have largely been described at the transcriptional level, with inhibition of FASN leading to upregulation of the *ets*-DNA-binding protein PEA3 – a negative regulator of *HER2* gene transcription – and consequent reduction in *HER2* mRNA expression.^82,126^ An additional layer of regulation involves the cellular localisation of HER2 in response to FASN levels and activity. Notably, small-interfering RNA (siRNA)-mediated silencing of FASN expression or chemical inhibition by using C75 markedly attenuates the membrane accumulation of HER2 and is also associated with broader alterations in cell morphology.^82^ Given that FASN is predominantly involved in the synthesis of saturated FAs, and these, in turn, modulate cell membrane dynamics including fluidity and lipid raft formation, inhibiting this lipogenic enzyme not only impairs the appropriate localisation of HER2 to the plasma membrane, but also impinges on the dimerisation of HER2 with epidermal growth-factor receptor (EGFR) that drives resistance to lapatinib and trastuzumab.^82,127^ Indeed, dual treatment with cerulenin and trastuzumab synergistically increases apoptosis in HER2-amplified breast cancer cells, indicating that FASN inhibition, and its associated effects on HER2 expression and localisation, could be an attractive combinatorial therapeutic target in this breast cancer subtype.^82^ Furthermore, as HER2-amplified tumours are particularly dependent on PI3K signalling, FASN could play a central role in modulating the initiation of growth-factor-dependent oncogenic signalling that is required for malignancy.^128–132^

### Metabolic regulation of the cancer epigenome

In addition to the modulation of pro-tumorigenic signalling networks, there is accumulating experimental evidence that FA metabolism exerts profound effects on the cancer epigenome, and this, in turn, regulates gene expression and cellular differentiation.^133^ A central role for ACLY and ACSS2 has been defined in this context as these enzymes are the major sources of acetyl-CoA, which is an essential cofactor for several histone-modifying enzymes.^134,135^ Activation of ACLY following phosphorylation on Ser^455^ by AKT directly promotes acetyl-CoA synthesis and increases global histone acetylation levels, even when nutrient availability is limiting.^136^ Under metabolically stressful conditions such as hypoxia, ACSS2 is also involved in the nuclear recycling of acetate to acetyl-CoA, and this, in turn, promotes acetylation of histones to increase the transcription of lysosomal and autophagy-related genes, which are essential for maintaining energy homoeostasis in cancer cells.^137^ Importantly, the sustained synthesis of acetyl-CoA by ACLY and ACSS2 maintains a histone acetylation profile that promotes the transcription of pro-proliferative and growth genes even under nutrient-deplete conditions, thus allowing cancer cells to more rapidly induce these tumorigenic genetic programmes when microenvironmental conditions become favourable.^134^

The availability of acetyl-CoA also regulates cell differentiation and stemness. This is particularly relevant in cancer, as the presence of a stem-cell niche is thought to contribute to the limitless replicative potential of a tumour, as well as restoring growth of lesions that arise from therapy relapse.^138^ In haematological malignancies, for instance, acetyl-CoA is an obligate cofactor for CREB-binding protein (CBP)/p300, which activates the transcription factor c-Myb and induces the expression of the self-renewal genes *Oct4*, *Sox2*, *Klf4* and *CSF1R*.^139^ The regulation of acetyl-CoA metabolic pathways also contributes to disease progression in pancreatic ductal adenocarcinoma (PDA), with oncogenic KRAS mutations co-operating with ACLY and ACSS2 activity to promote proliferation and metastasis.^140^ Acetyl-CoA-induced epigenetic remodelling in PDA creates a histone profile reminiscent of the foregut endoderm in the developing embryo, and is characterised by a transcriptional programme consisting of upregulation of pro-survival and metastatic genes, including *FOXA1, GATA5* and *Sox2*.^141^ Interestingly, metabolic crosstalk between ACLY–ACSS2 and the mevalonate pathway has also been observed in PDA, with acetyl-CoA serving as the predominant substrate for cholesterol and steroid synthesis.^140^ The metabolic products of the mevalonate pathway have well-characterised roles not only in the post-translational modification of oncogenes, including the farnesylation of Ras, but also on the epigenetic landscape through regulation of histone deacetylases (HDACs) and DNA methyltransferases (DNMTs).^142^ Importantly, inhibition of the mevalonate pathway with statins facilitates widespread effects on microRNA (miRNA) expression, reduced transcription of genes involved in folate metabolism such as dihydrofolate reductase (DHFR) as well as attenuation of HDAC activity leading to *CDKN1A* promoter acetylation and transcription of the tumour suppressor p21.^142^

Overall, the activation of lipogenic enzymes such as FASN, ACLY and ACSS2 are not just secondary events arising from hyperactive oncogenic signalling, but rather exist in a complex network involving reciprocal regulation. Furthermore, many of these enzymes generate metabolic by-products that exert profound effects on gene expression and whole-cell physiology, and their functions must, therefore, be considered in this broad context.

## Fatty acids support tumorigenesis and cancer progression

It is widely appreciated that FAs are essential to cancer cells because they sustain membrane biosynthesis during rapid proliferation, and provide an important energy source during conditions of metabolic stress. However, more intricate oncogenic roles of FAs and their by-products are beginning to be uncovered. These predominantly focus on the latter acting as signalling molecules that can directly regulate cellular homoeostasis, by modulating the surrounding microenvironment to create conditions that are conducive for tumour progression. The complex interplay of these multifaceted and diverse biological functions is discussed below.

### Membrane structure and fluidity

**Fig. 4:**
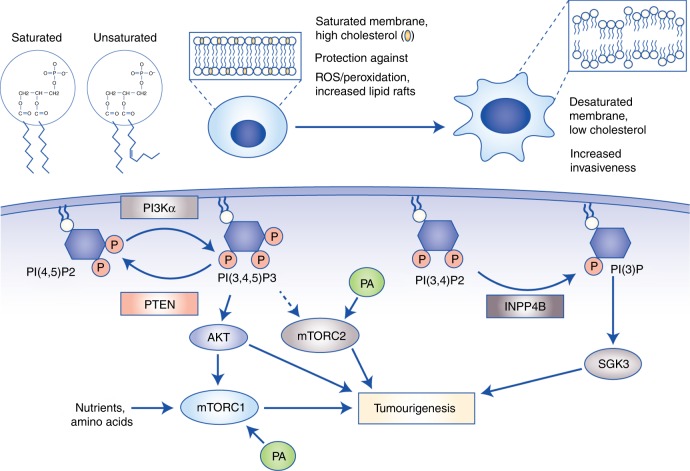
Fatty acids (FAs) regulate membrane architecture and oncogenic signalling pathways. **a** Membrane fluidity is largely determined by cholesterol levels and degree of FA desaturation. Cancer cells displaying elevated de novo lipogenesis can synthesise saturated phospholipids, which not only increases membrane rigidity, but also protects against peroxidation induced by reactive oxygen species. Conversely, highly migratory cells display more fluid membranes as a result of increased desaturation and cholesterol abundance, thus contributing to the epithelial-to-mesenchymal transition and metastasis. **b** FAs and their synthetic products also function as secondary messengers in signalling pathways, with the best characterised being the phosphoinositides. PI(3,4,5)P_3_ activates oncogenic AKT, and contributes to hyperactivation of mTORC1 and mTORC2. In addition, PI(3)P stimulates SGK3 that promotes tumorigenesis independently of AKT. Finally, phosphatidic acid can also directly bind to and activate the mTOR complexes. Abbreviations: PI3Kα, phosphoinositide 3-kinase α; PI(4,5)P_2_, phosphatidylinositol (4,5)-bisphosphate; PI(3,4,5)P_3_, phosphatidylinositol (3,4,5)-trisphosphate; PI(3,4)P_2_, phosphatidylinositol (3,4)-bisphosphate; PI(3)P, phosphatidylinositol 3-phosphate; mTORC, mammalian target of rapamycin complex; PA, phosphatidic acid; PTEN, phosphatase and tensin homologue; SGK3, serum- and glucocorticoid-induced protein kinase-3; INPP4B, inositol polyphosphate-4-phosphatase type II B.

FAs are essential building blocks for maintaining the structure and fluidity of the cell membrane. One of the advantages of the elevated rate of de novo lipogenesis in cancer cells is the synthesis of saturated and monounsaturated FAs, which are inherently more stable than polyunsaturated FAs because they contain fewer double bonds that can be targeted for peroxidation (Fig. 4a).^143^ Cancer cells with a higher degree of membrane saturation are less susceptible to oxidative stress induced by chemotherapeutic agents such as doxorubicin, whilst combinatorial inhibition of de novo lipid synthesis, either through siRNA knockdown of lipogenic enzymes or treatment with soraphen (an ACC inhibitor), synergistically increases cytotoxicity.^143^

Intracellular levels of cholesterol can also dramatically modulate membrane architecture that impacts cell migration and ultimately metastatic dissemination. Interestingly, incorporation of cholesterol in membranes generally reduces fluidity and consequently inhibits metastasis by limiting the capacity for a cell to change its shape, a process that is essential during the epithelial-to-mesenchymal transition (EMT) and intra-/extravasation from blood vessels (Fig. 4a).^144^ In support of this, cells undergoing EMT display increased cholesterol efflux through upregulation of the ATP-binding cassette transporter ABCA1, whilst human tumours overexpressing ABCA1 have strikingly higher rates of distant metastases.^144^ It is important to note, however, that increasing membrane rigidity can also be advantageous for cancer cells. This is particularly relevant for the development of multi-drug-resistant tumours, which typically contain lower levels of endogenously synthesised cholesterol and are consequently less permeable to anticancer agents.^145^

Epidemiological studies have highlighted controversial roles for aberrant cholesterol metabolism in cancer, thus raising important considerations for how tumours actually utilise cholesterol, and how it may be therapeutically exploited.^146^ For instance, elevated serum cholesterol levels are associated with increased incidence and recurrence of prostate cancer, whilst inhibition of its biosynthesis by using statins reduces colorectal, breast and endometrial cancer mortality.^147–149^ Conversely, numerous studies have shown either no association between cholesterol and cancer, or potential carcinogenic effects of statins.^150,151^ Therefore, resolving these disparate clinical outcomes remains a significant challenge and source of uncertainty for pursuing therapies targeting cholesterol metabolism. Perhaps insights can be gained from preclinical studies that consistently demonstrate a role for cholesterol in tumorigenesis, but in specific contexts. As discussed previously, during metastatic dissemination, cancer cells actively efflux cholesterol to maintain low membrane concentrations, thus promoting plasma membrane fluidity and EMT.^144^ In the case of advanced-stage disease, low cholesterol levels may, therefore, be advantageous for metastatic cells and contribute to cancer progression. On the other hand, the establishment of primary tumours are highly dependent on pro-proliferative and growth-stimulatory signalling pathways, and increased membrane cholesterol concentrations promote this through the formation of lipid rafts.^152^ Indeed, cholesterol-rich lipid rafts facilitate the accumulation of receptor tyrosine kinases, such as HER2 and IGF-1, to rapidly induce oncogenic signalling including PI3K–AKT.^153^ In this context, inhibiting cholesterol biosynthesis may be beneficial. Overall, in order to successfully exploit cholesterol metabolism as a therapeutic target in cancer, it may be necessary to first consider how the dependency of cancer cells on cholesterol changes with disease progression. With this in mind, actively lowering cholesterol in an advanced disease-stage setting may have limited or adverse effects as carcinoma cells present in metastases require low levels for EMT.^144^ Conversely, blocking cholesterol synthesis with statins could be more effective at inhibiting cancer initiation and proliferation at early disease stages when these tumours are more dependent on cholesterol for sustaining growth-factor-induced signalling.^153^

### Pro-tumorigenic signalling molecules

FAs are used for the synthesis of bioactive lipids that support cellular proliferation and survival by functioning as secondary messengers in signal transduction pathways. PtdIns are among the best-characterised signalling lipids, and are composed of two FA chains connected to an inositol ring and a glycerol backbone.^74^ The hydroxyl groups of the inositol ring can be phosphorylated at positions 3, 4 and 5 to generate several phosphoinositide species including monophosphorylated PI(3)P, PI(4)P and PI(5)P, diphosphorylated PI(3,4)P_2_, PI(3,5)P_2_ and PI(4,5)P_2_ and finally triphosphorylated PI(3–5)P3 (also known as PIP_3_).^74^ Of these, PIP_3_ is the most extensively studied because it facilitates the localisation of AKT to the plasma membrane, leading to its subsequent activation by phosphoinositide-dependent kinase 1 (PDK1) and mTORC2 downstream of hyperactive PI3K signalling (Fig. 4b). The multifaceted roles of AKT in oncogenesis have been alluded to earlier in this review: from a metabolic viewpoint, AKT stimulates glucose uptake by stabilising GLUT1/4, and triggers de novo lipogenesis and anabolic metabolism through mTORC1-dependent and -independent mechanisms.^62,84,89^ Moreover, AKT promotes survival by phosphorylating and inhibiting several pro-apoptotic proteins including BAD, procaspase-9 and the FOXO transcription factors, which positively regulate the expression of apoptotic enzymes.^154^ The regulation of oncogenic signalling by phosphoinositides can also be mediated through the activity of lipid phosphatases such as PTEN and inositol polyphosphate-4-phosphatase type II (INPP4B). PTEN in particular has been the subject of extensive study, as it acts as the main negative regulator of PI3K signalling by dephosphorylating PIP_3_ to PIP_2_.^74^ Conversely, other phosphatases involved in phosphoinositide metabolism can activate PI3K signalling independently of AKT. For instance, the sequential conversion of PIP_3_ into PI(3,4)P_2_ and PI(3)P by Src homology 2-containing inositol 5-phosphatase (SHIP) and INPP4B, respectively, activates SGK3 (Fig. 4b).^155–157^ PI3K–SGK3 signalling initiated by PI(3)P and INPP4B drives cell proliferation, invasion and tumour growth, demonstrating that phosphoinositides functioning as secondary messengers can dynamically regulate the initiation of different signalling axes, thus providing compensatory pro-tumorigenic effects.^155,156^

Since PtdIns are the precursors of phosphoinositides, their spatial and temporal regulation are essential in regulating several cellular processes. Consequently, features associated with normal FA metabolism including localisation and transport of PtdIns, as well as their enzymatic conversion into various phosphoinositide species, are often exploited by cancer cells to fuel pro-proliferative signalling. In terms of co-ordinating the spatial localisation of PtdIns pools – and by extension their downstream conversion into phosphoinositide secondary messengers – PtdIns transfer proteins (PITPs) function as central regulators.^158^ An important feature of PITPs is their capacity to transfer PtdIns between subcellular compartments in an ATP-independent fashion, thereby efficiently localising PtdIns that are initially synthesised in the ER to other membranes with comparably lower PtdIns concentrations, such as the plasma membrane.^159^ A specific subclass of PITPs are type 1 START-like PITPs that encompass three main isoforms: PITPα, PITPβ and RdgBβ.^159^ PITPα is arguably the best-characterised isoform, and most widely associated with disease pathology due to its high affinity for PtdIns binding.^159^ For instance, PITPα is required for normal neuronal development by regulating the co-ordinated accumulation of PtdIns to the leading edge of axons, thus ultimately contributing to localised PIP_3_ generation, by PI3K-enhanced AKT signalling, and axon elongation.^160^ An integral role for PITPα in promoting signal transduction pathways initiated by RTKs has also been described in the context of EGFR activation, suggesting that PITPα could also be closely associated with oncogenic signalling.^161^ This probably involves the PITPα-mediated provision of PtdIns to the plasma membrane, leading to the localised accumulation of a phosphoinositide pool at activated RTKs and downstream synthesis of mono-, di- and triphosphorylated phosphoinositide secondary messengers.^161^ Interestingly, PITPα has also been implicated in tumour metastasis through an intricate mechanism linking PIP_2_ and inositol 1,4,5-triphosphate (IP_3_) signalling in platelet cells.^162^ In this model, PIPTα drives the formation of a specific pool of PIP_2_ that is utilised for IP_3_ synthesis through enhanced phospholipase Cγ (PLCγ) activity.^162^ A major implication of this PIPTα–PIP_2_–IP_3_ signalling axis is the production of thrombin by platelets, allowing them to adhere to circulating tumour cells and facilitating their dissemination to distant tissues.^162^ Overall, these findings illustrate that dysregulated FA metabolism in the form of aberrant transport and localisation of lipid molecules, namely PtdIns, has notable effects on the spatial production of secondary messengers that ultimately impact on cell-signalling pathways.

In terms of phosphoinositide metabolism, growth-factor stimulation promotes the synthesis of PIP_3_ within seconds, leading to the rapid induction of PI3K–AKT signalling. This implies the existence of a highly co-ordinated enzymatic system that can efficiently catalyse the conversion of membrane-localised pools of PtdIns into pro-tumorigenic phosphoinositides. Indeed, the scaffold protein IQGAP1 is essential in this process, as it can simultaneously bind PI4KIIIα, PIPKIα and PI3K to facilitate the sequential synthesis of PI(4)P, PI(4,5)P_2_ and PI(3,4,5)P_3_ from PtdIns, respectively.^163^ It is well accepted that PIP_2_ is present in excess of up to 100-fold when compared with PIP_3_ at the plasma membrane, even in stimulated cells.^164^ Therefore, the spatial regulation of PIP_3_ generation mediated by IQGAP1 – which, in turn, is dependent on the presence of localised pools of PIP_2_ and PtdIns at sites of activated RTKs – could represent the rate-limiting step in the efficient synthesis of this secondary messenger, as opposed to the general supply of FAs per se.^163^

In addition to PtdIns, PAs can serve as potent signalling molecules. The predominant source of PAs is from the hydrolysis of PC and PE by phospholipase D 1/2 (PLD1/2); however, glycerol-3-phosphate (G3P) can also be used as a substrate for glycerol-3-phosphate acyltransferase (GPATs) to synthesise PA.^165^ Notably, PAs can directly bind and stabilise mTOR, thus leading to increased activity of both mTORC1 and mTORC2.^166^ Inhibition of PA synthesis reduces mTORC2-dependent phosphorylation of AKT on Ser^473^, suggesting that mTOR, in addition to sensing nutrients including amino acids,^167,168^ also integrates signals from lipids to ultimately coordinate cellular growth and proliferation.^169^ It is particularly interesting to note that PAs can directly compete with mTOR inhibitors such as rapamycin for binding to the mTOR complexes.^169^ This finding may have important clinical implications, as it suggests that the levels of PA and the activity of enzymes involved in its synthesis, such as PLD and GPAT, could influence sensitivity to mTOR inhibition.^169^

**Fig. 5:**
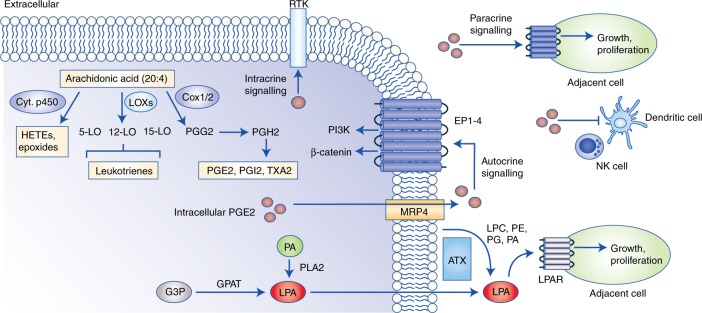
Remodelling of the tumour microenvironment by bioactive lipids. Eicosanoids and lysophosphatidic acid species can be secreted into the surrounding microenvironment and stimulate cell proliferation through both autocrine and paracrine mechanisms. Arachidonic acid (AA) serves as the main precursor for eicosanoid synthesis, and these include pro-inflammatory prostaglandins, leukotrienes and eicosatetraenoic acids. PGE2 can stimulate cell proliferation through autocrine and paracrine signalling, and this is largely mediated through the activation of EP1–EP4 receptors. Moreover, prostaglandins contribute to immunosuppression by attenuating the activation of natural killer, dendritic and cytotoxic T cells. Lysophosphatidic acids are also relevant signalling molecules, and can be produced intracellularly from glycerol-3-phosphate or phosphatidic acid, or extracellularly through the enzyme autotaxin, which uses existing phospholipids, such as phosphatidylglycerols and phosphatidylethanolamines as substrates. Similar to eicosanoids, lysophosphatidic acids exert their tumorigenic effects by binding to the LPAR family of G-protein-coupled receptors. Abbreviations: RTK, receptor tyrosine kinase; LOX, lipoxygenase; COX, cyclo-oxygenase; PGG2, prostaglandin G2; PGH2, prostaglandin H2; PGE2, prostaglandin E2; PGI2, prostaglandin I2; TXA2, thromboxane A2; HETE, hydroxytetraenoic acid; EP, prostaglandin E2 receptor; NK, natural killer cell; G3P, glyceraldehyde 3-phosphate; GPAT, glycerol-3-phosphate acyltransferase; PA, phosphatidic acid; PLA2, phospholipase A2; LPA, lysophosphatidic acid; LPAR, lysophosphatidic acid receptor; ATX, autotaxin.

Bioactive lipids can also stimulate cell proliferation through autocrine and paracrine mechanisms that require the activation of G-protein-coupled receptors (GPCRs) (Fig. 5). Among the most potent of these lipid species are lysophosphatidic acids (LPAs) consisting of a phosphate head group attached to a glycerol backbone and a single FA tail.^170^ The production of LPA is influenced by the abundance of various lipid species. For instance, LPA can be generated through two main mechanisms: the first involves cleavage of existing phospholipids at the sn-2 position by phospholipases (PLAs) to release a lysophospholipid and a FA, and the second requires the lysophospholipase D activity of autotaxin to convert LPC into LPA extracellularly.^171^ In terms of the first mechanism in particular, cells must have increased synthesis of PA that specifically releases LPA following cleavage by phospholipases. In this context, a dominant role for PLD2 has been described, with gastric, breast and colorectal cancers displaying elevated protein expression and activity.^172^ Moreover, DAG can directly contribute to the cellular pool of PA through the enzymatic activity of DAG kinases (DGKs).^173^ Notably, DGKα has been shown to promote the synthesis of PA from DAG, which can subsequently be used to generate LPA in fibrotic tissues, thus negatively impacting the efficacy of radiation therapy.^174^ Therefore, dysregulated lipid metabolism can directly regulate the generation of LPA by modulating the cellular pool of PA. The latter is also activated by CDP to form CDP–DAG that can then be converted into glycerophospholipids PtdIns, PG and cardiolipin. Importantly, this biosynthetic pathway demonstrates how changes in LPA and/or PA might play a crucial role in regulating intracellular signalling and trafficking by affecting the pool of PI, which acts as the major precursor of all phosphoinositides.

Several LPA species containing FA tails of varying carbon length and desaturation exist, with the most common and biologically relevant being LPA(16:0) and LPA(18:1).^175^ Human cells express six LPA receptor (LPAR) genes encoding LPAR1–6 to which LPAs bind and exert their pro-tumorigenic effects.^170^ These include activation of PI3K and Ras–MAPK signalling that have well-established roles in driving cell proliferation by inhibiting negative regulators of cell-cycle progression such as p21, p27 and p15, as well as induction of RHO GTPases that promote cell migration through remodelling of the actin cytoskeleton.^176–178^ LPAs are also implicated in autocrine and paracrine-signalling networks, thus serving as important intermediaries between tumour cells and the microenvironment. Indeed, in pancreatic ductal adenocarcinomas (PDAC), pancreatic stellate cells (PSCs) and cancer-associated fibroblasts (CAFs) display elevated synthesis of LPCs from glucose and glutamine, and ultimately release these lysophospholipids into the surrounding microenvironment.^179^ PDAC cells display increased secreted levels of autotaxin, facilitating the rapid and localised conversion of LPC into LPA, and consequent activation of AKT signalling through stimulation of LPAR1/2.^179^ Pharmacological inhibition of autotaxin significantly reduces PDAC tumour growth in vivo, and this effect is more pronounced following co-transplantation with PSCs, thus highlighting the importance of the latter in dynamic lipid remodelling through local LPC release and autotaxin-mediated conversion into LPA.^179^ These results also suggest that therapeutically targeting metabolic interdependencies between the primary tumour and the surrounding microenvironment could be an effective strategy for the treatment of PDAC.^179^

### Eicosanoids remodel the tumour microenvironment

An important subclass of bioactive lipid molecules are eicosanoids. The omega-6 FA arachidonic acid (AA) serves as the main precursor of several eicosanoid species, including prostaglandins, thromboxanes and leukotrienes, each of which has pro-inflammatory and pro-tumorigenic effects.^72^ There are three main mechanisms through which cancer cells can obtain AA: (1) direct exogenous uptake by using specific transporters, such as CD36 and TWIK-related AA-stimulated K^+^ (TRAAK) channels, (2) de novo synthesis from linoleic acid (LA) and (3) cleavage of the sn-2 position of existing membrane phospholipids through the enzymatic activity of phospholipases. Each of these processes has been extensively reviewed previously.^72,180^ The predominant enzymes involved in prostaglandin production are the prostaglandin G/H synthetases COX1 (PTGS1) and COX2 (PTGS2). The former is constitutively active and regulates normal cellular processes including angiogenesis and blood clotting, whilst COX2 expression is selectively induced by growth factors and chemokines, and is, therefore, more closely linked with inflammation.^72^ The cyclo-oxygenase and peroxidase activities of COX enzymes sequentially convert AA into prostaglandin G2 (PGG2) and prostaglandin H2 (PGH2), respectively.^72^ Other eicosanoids including PGE2, PGD2, prostacyclins and thromboxanes can be derived from PGH2.^72^

Prostaglandins exert pro-inflammatory and pro-tumorigenic effects in both autocrine and paracrine fashions by activating G-protein-coupled prostanoid receptors including EP1–4, DP1, PGF receptor (FP), PGI receptor (IP) and TX receptor (TP) (Fig. 5).^181^ Tumour-initiating events triggered by prostaglandins include PGE2-mediated activation of PI3K signalling and induction of ERK following binding to the EP4 receptor, thus stimulating proliferation, as well as cell migration through stabilisation of β-catenin and c-MET induction.^182,183^ In addition, in models of colorectal cancer, intracellular PGE2 can induce phosphorylation and activation of EGFR, leading to induction of MMPs and a consequent promotion of cell invasiveness.^184^ Prostaglandins can also promote adaptation to microenvironmental stress conditions such as hypoxia.^185^ Under hypoxic conditions, HIF1α induces the expression of COX2, leading to a concomitant overproduction of PGE2 that stimulates tumour angiogenesis in a VEGF- and chemokine (c–X–c motif) ligand 1 (CXCL1)-dependent manner.^186,187^ It is interesting to note that at least in colorectal cancer, HIF1 can actually induce cell-cycle arrest by binding to β-catenin and displacing transcription factor 4 (TCF4), thereby blocking the formation of a functional β-catenin–TCF4 transcriptional complex and inhibiting the transcription of genes involved in proliferation.^185,188^ This mechanism is particularly intricate because it demonstrates how the crosstalk between HIF and prostaglandin signalling can induce a ‘hibernation’ state in cancer cells that is characterised by sustained nutrient acquisition through vascularisation, and reduced energy expenditure.^185,188^ The main implications of this could be that cancer cells are better equipped to recover from hypoxia and resume proliferation immediately following reoxygenation.

Further to the direct effects on tumour cells, prostaglandins can also modulate the surrounding microenvironment, and this has been extensively demonstrated in the context of inhibition of the anti-tumour immune response (Fig. 5). One mechanism through which elevated PGE2 promotes tumour immune evasion is by abrogating co-stimulation and complete activation of CD8^+^ T lymphocytes mediated by binding of intercellular adhesion molecule 1 (ICAM-1) expressed on tumour cells and the lymphocyte receptor LFA-1.^189^ In addition, accumulation of conventional type 1 dendritic cells (cDC1s) is essential for initiating the anti-tumour immune response, and requires the infiltration of natural killer cells within the tumour microenvironment.^190^ However, in models of BRAF^V600E^ mutant melanomas, overproduction of COX2-derived PGE2 directly inhibits the production of CXCL1 and chemokine (c–C motif ligand 5 (CCL5) chemokines by natural killer (NK)) cells, thereby attenuating the migration of cDC1s to the tumour site.^190^ Importantly, the accumulation of NK and cDC1 cells is associated with better prognosis in melanoma and breast cancers, indicating that the immunomodulatory properties of prostaglandins could have significant clinical implications.^190^ Indeed, the response of melanomas harbouring BRAF^V600E^ mutation to anti-programmed cell death protein 1 (PD1) immune checkpoint inhibitors is significantly improved following combinatorial therapy with COX inhibitors including aspirin and celecoxib.^191^

An additional pathway for AA metabolism is its conversion into arachidonyl-CoA following the ligation of acetyl-CoA catalysed by the acyl-CoA synthetase ACSL4.^192^ Arachidonyl-CoA can be subsequently esterified to form TAGs and incorporated into phospholipids, or utilised as a substrate by COX2 to enhance eicosanoid biosynthesis.^193^ ACSL4 is also implicated in the localised release of AA in the mitochondria, and this requires the transport of arachidonyl-CoA to the inner mitochondrial membrane via the translocator protein (TSPO) and hydrolysis by acyl-CoA thioesterase 2 (ACOT2).^192^ Importantly, several studies have implicated ACSL4 in tumorigenesis, with advanced-stage breast, colorectal, hepatocellular and prostate carcinomas displaying increased expression at both the mRNA and protein levels.^192^ The oncogenic effects of elevated ACSL4 are twofold. Firstly, mitochondrial AA that accumulates as a consequence of ACSL4 activity is predominantly directed towards the biosynthesis of leukotrienes including 5-, 12- and 15-hydroxyeicosatetraenoic acid (HETE).^194^ These metabolites potently activate leukotriene B4 receptors and potentiate several oncogenic signalling pathways including PI3K–AKT and Wnt–β-catenin, thus promoting cell migration and proliferation in breast and prostate cancer cells.^194^ Secondly, the excessive accumulation of unesterified polyunsaturated FAs (PUFAs) promotes apoptosis through induction of the ER-stress response, activation of caspase-3 and tumour necrosis factor α (TNFα) signalling.^195^ ACSL4 limits the cytotoxicity associated with elevated cellular pools of unesterified AA by producing arachidonyl-CoA, thereby increasing the apoptotic threshold and survival of castration-resistant prostate cancer (CRPC) cells.^192^ It is important to note, however, that the localised accumulation of arachidonyl-CoA and AA in the mitochondria can also contribute to membrane depolarisation and electron transport chain uncoupling, leading to increased ROS production.^196^ If left unchecked, the elevation in ROS levels can increase lipid peroxidation and cell death induced by ferroptosis.^196^ Thus, cancer cells must also have adequate anti-oxidative responses, including increased Nrf2 activity and NADPH/glutathione biosynthesis, to capitalise on the pro-tumorigenic effects of ACSL4 overexpression.

## Therapeutically exploiting fatty acid metabolism in cancer

Given the extensive role of FAs in cancer pathogenesis, there is substantial clinical interest in developing therapies that target FA metabolic reprogramming. The majority of inhibitors designed for this purpose target specific enzymes involved in de novo FA synthesis and exogenous lipid uptake (Table 1); however, there is also a resurgent interest in understanding how specific dietary interventions may synergistically improve the efficacy of existing cancer therapeutics. Current strategies and considerations for successfully targeting the tumour lipidome are discussed below.

**Table 1:** Non-exhaustive list of therapies targeting lipid metabolism as cited in the main text.

Target	Drug	Drug development stage	Disease type
FASN	C75	Preclinical	Breast cancer^197,198^
	Cerulenin	Preclinical	Breast cancer^197,198^
	Orlistat	Preclinical	Breast cancer^198^
		FDA-approved (marketed as Alli and Xenical)	Obesity management
	TVB-3166	Preclinical	Colorectal, breast cancer^202,203^
	TVB-2640	Phase 2 clinical trials	Breast cancer (ID: NCT03032484, NCT03179904 and NCT02223247)^205^
ACLY	SB-204990	Preclinical	Lung, prostate cancer^34^
	Hydroxycitrate	Randomised controlled trial	Hypercholesterolaemia and obesity^210,211^
	ETC-100 (dual ACLY inhibitor/AMPK activator)	Phase 2/3 clinical trials	Hypercholesterolaemia (ID: NCT01941836 and NCT03001076)^207–209^
ACSS2	N-(2,3-di-2-thienyl-6-quinoxalinyl)-N’-(2-methoxyethyl)urea	Preclinical	Bladder cancer^217,218^
SCD	SSI-4	Preclinical	Hepatocellular carcinoma^219^
	Betulinic acid	Preclinical	Colorectal cancer^220^
	MF-438	Preclinical	Lung cancer^221^
Whole-body metabolism	Omega-3 FA supplementation	Double-blind randomised control trial	Rheumatoid arthritis^227^
	Omega-3 FA supplementation	Phase 1 clinical trial	Breast cancer (ID: NCT0182580)
	Omega-6:omega-3 FA ratios of 46:1, 10:1 and 1.3:1	Preclinical	Prostate cancer^224^

*FASN* fatty acid synthase, *ACLY* ATP–citrate lyase, *ACSS2* acetyl-CoA synthetase 2, *SCD* stearoyl-CoA desaturase

### Targeting FASN: a challenging past but promising future

In terms of therapeutically targeting dysregulated lipid metabolism in cancer, FASN has arguably received the most widespread interest, and this is not surprising given its multifaceted roles in supporting both anabolic metabolism and oncogenic signalling. However, the transition of FASN inhibitors from bench to bedside has largely been elusive, and marked with several challenges and shortcomings. This is particularly relevant for the first-generation FASN-targeting drugs, such as C75, orlistat and cerulenin. These compounds initially showed great promise in preclinical studies, with both significantly reducing tumour xenograft growth and inducing cell-cycle arrest, whilst also sensitising breast cancer cells to ROS-inducing chemotherapies by decreasing the synthesis of saturated lipids.^102,197,198^ In spite of these successful outcomes in the laboratory, significant clinical obstacles included detrimental systemic side effects characterised by drastic weight loss and anorexia,^199^ thus highlighting the involvement of FA metabolic enzymes in both disease pathology and normal whole-body metabolic homoeostasis. Indeed, recent studies have demonstrated that in both normal and malignant ovarian models, *FASN* expression largely reflects the proliferative and growth state of a cell, rather than malignancy per se.^200^ Interestingly, proliferative non-malignant ovarian surface epithelial (OSE) and fallopian tube secretory epithelial (FT) cells express levels of FASN comparable with ovarian cancer cell lines, and display similar sensitivities to FASN inhibition by C75 and G28UCM.^200^ These observations, however, do need to be reconciled with the commonly held notion that elevated de novo lipogenesis driven by FASN is a metabolic hallmark of cancer, but not normal cells, and therefore, the latter should be largely insensitive to FASN inhibition. Previous studies demonstrating that FASN-catalysed endogenous FA synthesis in normal liver and adipose tissue is actually stimulated by a high-carbohydrate diet not only add another layer of complexity to this notion, but also suggest that monitoring nutrient availability may be indispensable in determining the whole-body sensitivity of noncancerous cells to FASN inhibition.^201^ Thus, in terms of clinical implications, it will be important to characterise FASN as a potential general proliferative marker – rather than being solely associated with malignancy – as well as understanding the effects of nutrition on the dependence of normal tissues for FASN-driven lipogenesis.

More recently, next-generation FASN inhibitors, including TVB-3166 and TVB-2640, have shown tremendous anti-tumour potential in preclinical breast and colorectal cancer models, as well as excellent tolerability and limited systemic toxicity in early-phase clinical trials.^202,203^ One explanation for the improved tolerance of the next-generation inhibitors could be that in contrast to C75 and cerulenin, TVB-3166 and TVB-2640 do not contribute to the indirect activation of CPT1 in peripheral tissue.^202^ The induction of β-oxidation peripherally following C75 or cerulenin treatment contributes to increased energy expenditure, loss of adipose tissue and significant weight loss, whereas next-generation inhibitors display higher specificity for FASN with limited off-target effects.^202,204^ Accompanying the development of novel FASN therapies is a concerted effort to better stratify patients who will actually benefit from FASN inhibition. A potential strategy for this endeavour may include assessment of HER2 status, particularly in light of the connections between FASN expression and signalling downstream of HER2, which have been discussed earlier in this review.^82^ Indeed, there are now two clinical trials in Phase 2 stages evaluating the combinatorial effects of TVB-2640 and chemotherapy in HER2-positive breast cancer (Clinical Trial ID: NCT03179904) and astrocytomas (Clinical Trial ID: NCT03032484).^205^

### Targeting ACLY and ACSS2: limiting the metabolic substrates for lipogenesis

Increased expression and activity of ACLY is observed across several tumour types including glioblastoma, colorectal, breast and hepatocellular carcinomas.^34^ Considering its role in generating acetyl-CoA, which is the main substrate for lipogenesis and cholesterol synthesis, targeting ACLY has proven to be an effective therapy for treating hypercholesterolaemia and hyperlipidaemia.^206^ Importantly, several ACLY inhibitors including ETC-1002 (Phase 2/3 clinical trials) and hydroxycitrate (randomised control trials) have shown high efficacy in lowering low-density lipoprotein–cholesterol and good tolerability in patients with cardiovascular disease and type 2 diabetes (Table 1), thus indicating that ACLY inhibition could represent a well-tolerated therapeutic strategy.^207–211^

There is considerable interest in ACLY as a target for anticancer drugs; however, clinical trial studies in this context are far more limited when compared with dyslipidaemia cases. Nevertheless, there is a plethora of preclinical evidence to support the integral role of ACLY in tumorigenesis and the potential clinical benefits for its selective inhibition. In particular, both genetic and pharmacological targeting of ACLY significantly reduces the growth of lung and prostate tumour xenografts, and this anti-tumorigenic effect is more pronounced in highly glycolytic cells.^34^ Perhaps this is not surprising given that ACLY largely generates acetyl-CoA from glucose-derived citrate, but it does suggest that metabolic stratification of tumours may improve the potency of ACLY inhibitors such as SB-204990 and hydroxycitrate.^34^ This is particularly relevant when one considers the current limitations of ACLY-targeted cancer therapeutics, which predominantly include the relatively high drug concentrations required for complete inhibition of ACLY activity. Thus, by identifying tumours with high rates of glucose consumption that are consequently more dependent on ACLY activity for glycolysis-fuelled lipogenesis, dosing regimes with lower drug concentrations could be implemented.^34^ Furthermore, structural studies focussing on characterising the protein domains of the ACLY tetramer have revealed novel hydrophobic regions near the citrate-binding site that have untapped potential for improving the drugability of ACLY.^212^

Another approach for implementing ACLY inhibition as a therapeutic strategy for cancer is to consider its role in signalling networks that regulate cellular responses to energy stress. For instance, in CRPC cells, inhibition of ACLY disrupts ER and energy homoeostasis in CRPC cells, leading to AMPK activation and sensitisation to androgen receptor inhibitors.^213^ Intriguingly, supplementation of exogenous FAs is sufficient to restore hormone resistance in CRPC cells by alleviating the ER and energy stress instigated by ACLY inhibition, demonstrating the importance of an ACLY–AMPK network in regulating energy homoeostasis that could be exploited pharmacologically.^213^ Indeed, several AMPK-activating therapies such as metformin have shown anticancer and anti-inflammatory properties, and importantly are well tolerated in patients.^214^ Notably, some ACLY inhibitors currently used for the treatment of hypercholesterolaemia, such as ETC-1002, are also dual activators of AMPK, and although not yet investigated, it would be interesting to assess the applicability of these drugs in the cancer therapy context.^207^ While dual targeting of ACLY and AMPK does seem like an attractive therapeutic strategy, it is necessary to acknowledge the instances in which the latter may actually promote tumorigenesis. For instance, the presence of functional LKB1 is essential for complete AMPK activation, and given that this kinase is frequently mutated – including in 34% of lung carcinomas and 20% of cervical cancers – it may be necessary to first stratify patients based on LKB1 status to evaluate the true therapeutic potential of AMPK activators.^215^ Furthermore, the extent of mTORC1 hyperactivation should also be considered, as tumours that are dependent on mTOR signalling are more likely to have a better response to AMPK activation.^214^ Closely linked with this idea are the regulatory feedback mechanisms that exist between mTORC1 and mTORC2–AMPK is a well-characterised negative regulator of mTORC1; however, the potential compensatory effects leading to mTORC2 hyperactivation and initiation of pro-tumorigenic signalling, such as induction of AKT, must be carefully considered.^214^ Finally, AMPK activation promotes calcium-induced migration of prostate cancer cells through calmodulin-dependent protein kinase (CaMKKB) signalling.^216^ Thus, it appears that therapeutically exploiting ACLY and AMPK activities requires extensive molecular characterisation of a patient’s tumour, and may be context-dependent.

Although the effects of ACLY inhibition predominantly focus on FA synthesis, an additional biological feature of reducing acetyl-CoA synthesis is remodelling histone acetylation and gene expression patterns.^135^ Given the essentiality of acetyl-CoA in other metabolic pathways and gene regulation, it may be necessary to undertake parallel transcriptomic and metabolomic profiling of adjacent normal tissue in order to fully ascertain the systemic consequences of inhibiting acetyl-CoA production. To improve the selective modulation of acetyl-CoA production in tumour cells, whilst simultaneously sparing healthy tissue, an alternative strategy could be to exploit therapeutic windows arising from the plasticity of cancer cells in metabolically challenging microenvironments. With regard to acetyl-CoA metabolism, tumours specifically upregulate ACSS2 under conditions of hypoxia or lipid deprivation, leading to increased acetate uptake and ligation with CoA to produce acetyl-CoA for de novo lipogenesis.^33^ Importantly, a HIF–SREBF2 signalling axis drives ACSS2 expression and confers exquisite sensitivity to its inhibition in hypoxic or otherwise metabolically stressed tumours.^33^ Although the development of ACSS2-specific inhibitors is lagging considerably behind ACLY-targeted therapies, efforts are being made to bridge this gap through identification of novel selective inhibitors by using high-throughput screens.^217^ In fact, one of the most potent compounds identified, N-(2,3-di-2-thienyl-6-quinoxalinyl)-N'-(2-methoxyethyl)urea, has already been shown to sensitise chemotherapy-resistant bladder cancers that are dependent on acetate metabolism to cisplatin.^217,218^

### Targeting SCD: inhibiting fatty acid desaturation

Desaturation of FAs by SCD enzymes produces monounsaturated FAs that contribute to the synthesis of additional lipid species including glycerophospholipids and sphingolipids.^56^ Ectopic SCD expression promotes EMT and is associated with poor prognosis in breast and colorectal cancer.^56^ This provides a therapeutic opportunity for tumours that depend on canonical SCD-mediated desaturation, and the effect is more apparent in the absence of exogenous lipids.^55^ SCD inhibitors, such as SSI-4, betulinic acid (BetA) and MF-438, have been shown to induce apoptosis in cancer cells through multiple mechanisms including induction of the ER-stress response, modulating mitochondrial dynamics by altering cardiolipin structure, and growth inhibition of cancer stem cells (Table 1).^55,56,219–221^ Interestingly, not all cancers display sensitivity to SCD inhibition, as they rely on a compensatory desaturation pathway by utilising FADS2 to generate sapienate from palmitate.^57^ In this context, sapienate, instead of palmitoleate generated from SCD, contributes substantially to membrane synthesis in hepatocellular and lung carcinomas.^57^ As a result, a significant reduction in tumour area is only observed following combinatorial treatment of hepatocellular carcinoma xenografts with SCD and FADS2 inhibitors, thereby blocking any compensatory pathways for obtaining desaturated FAs.^57^ In light of these findings, it is important to consider that the canonical function of FADS2 is the desaturation of LA to γ-linolenic acid (C18:3n6).^180^ Given that FADS2 is obligatory for the de novo synthesis of long-chain omega-6 FAs including AA, it would be interesting to further investigate the potential regulatory mechanisms and microenvironmental conditions that determine the substrate preference of FADS2. This could yield significant insight into the interplay of sapienate metabolism with other FA biosynthetic pathways, and in turn uncover additional compensatory mechanisms that can be exploited therapeutically.

### Dietary interventions and cancer therapeutics

Finally, there has been a resurgent interest in studying the role of dietary interventions in cancer therapy.^76,222,223^ For instance, it has been demonstrated that a low-carbohydrate ketogenic diet dramatically increases the efficiency of PI3K inhibitors and synergistically reduces the growth of *PIK3CA-*mutant tumours.^76^ Since humans can only obtain essential omega-3 and omega-6 FAs from the diet, it is tempting to speculate that dietary modifications based on lipid consumption could also have an impact on tumorigenesis. Omega-3 FAs, including eicosapentaenoic and docosahexaenoic acids (EPA and DHA, respectively), are widely accepted to have anti-inflammatory properties by competing with AA for COX2 binding, and subsequently producing PGE3 instead of PGE2.^224^ In contrast, omega-6 FAs, such as LA and AA, are the precursors for pro-inflammatory eicosanoids.^225^ The recommended dietary ratio of omega-6:3 FAs is 1:1; however, the Western diet, which is significantly enriched in omega-6s, has a ratio of 15:1,^225^ and this has significant implications for the progression of several cancers including breast and colorectal.^226^ Conversely, a diet rich in omega-3 FAs has been associated with the pathological improvement of several inflammatory diseases, such as arthritis and asthma^224,227,228^ (Table 1), as well as reduced risks of developing breast, colorectal and prostate cancers.^224,229^ It is important to note, however, that excessive omega-3 consumption can also have undesirable side effects including immunosuppression.^230^ Therefore, it is essential that careful optimisation of omega-3:omega-6 ratios is undertaken for each patient. By taking these factors into account, modulation of dietary fat intake either alone or in combination with existing therapies could have tremendous potential in therapy response.

## Conclusions and future perspectives

It is now widely appreciated that cancer cells display significant rewiring in their FA metabolism. This is not surprising when one considers the intricate regulation of lipid homoeostasis in the context of oncogenic signalling pathways, such as PI3K–AKT–mTOR and their diverse cellular functions. These are not just limited to cell intrinsic processes, such as membrane synthesis or serving as intracellular secondary messengers, but also extend to remodelling of the entire tumour microenvironment through paracrine-signalling mechanisms. In terms of therapeutic strategies, it is unlikely that inhibiting single enzymes or pathways will be sufficient to harness the full potential of targeting FA metabolism in cancer treatment. Instead, it is necessary to consider the complex framework within which FAs and their by-products are synthesised and exert their functions, including the activation of various compensatory pathways that sustain FA metabolism, and dynamic interactions with the tumour microenvironment and nutrient availability. This area of research holds great promise for the implementation of novel combinatorial strategies that exploit the unique dependency of cancer cells on FAs, both through pharmacological inhibition of metabolic targets and dietary interventions.

## Data Availability

Not applicable.
